# Interpretable machine learning-based prediction of mortality in critical cancer patients with delirium: A retrospective cohort study

**DOI:** 10.1016/j.apjon.2025.100760

**Published:** 2025-07-19

**Authors:** Yang He, Ning Liu, Sicheng Hao, Mimei Xu, Yingchun Zeng

**Affiliations:** a Department of Emergency Medicine, Sir Run Run Shaw Hospital, Zhejiang University School of Medicine, Hangzhou, Zhejiang Province, China; b Institute for Medical Engineering and Science, Massachusetts Institute of Technology, Cambridge, MA, USA; c Health Management Center, Quzhou KeCheng People's Hospital, Quzhou, China; d Alice Lee Centre for Nursing Studies, Yong Loo Lin School of Medicine, National University of Singapore, Singapore

**Keywords:** Delirium, Cancer patients, Mortality, Machine learning, Shapley additive explanations

## Abstract

**Objective:**

Delirium in cancer patients presents a significant clinical challenge, often leading to increased mortality, prolonged hospital stays, and higher healthcare costs. This study aimed to develop an interpretable and generalizable machine learning (ML) model for early prediction of mortality risk in cancer patients with delirium.

**Methods:**

A retrospective cohort study design was employed, utilizing data from the Medical Information Mart for Intensive Care IV (MIMIC-IV) database. Five ML models were subsequently constructed and evaluated.

**Results:**

A total of 1893 cancer patients with delirium were included in the analysis, of whom 685 (36.2%) died within 28 days who were admitted to the intensive care unit at Beth Israel Deaconess Medical Center between 2008 and 2022. The Category Boosting (CatBoost) algorithm outperformed other ML models, achieving the highest area under the curve (AUC) on both training and validation datasets. Its robustness was supported by a bias-corrected performance curve closely aligned with the ideal line and the greatest net benefit in decision curve analysis across all threshold probabilities (0–1). The top five predictors of 28-day mortality were high Glasgow Coma Scale and Acute Physiology and Chronic Health Evaluation II scores, use of antibiotics, propofol, and vasopressors.

**Conclusions:**

This study developed an optimal and explainable ML model for predicting 28-day mortality in cancer patients with delirium. The CatBoost algorithm demonstrated stable and robust performance, and interpretability analysis highlighted key predictors. These findings may aid early clinical decision-making and targeted interventions for this high-risk population.

## Introduction

Delirium, a neurocognitive syndrome characterized by acute disturbances in attention, cognition, and consciousness, poses a significant challenge in the care of hospitalized cancer patients.^1^, ^2^, ^3^ Its prevalence is substantial, ranging from 10.3% to 34.3% across various studies,^2^, ^3^, ^4^ and its consequences are severe. Patients experiencing delirium often suffer from prolonged hospital stays, increased risk of falls and other complications, higher healthcare costs, and increased mortality.^3^, ^4^, ^5^, ^6^

A variety of risk factors contribute to its development, including older age, lower educational attainment, history of hypertension, alcohol abuse, higher age, Acute Physiology and Chronic Health Evaluation (APACHE) II scores, and the administration of sedative and analgesic medications.^7^, ^8^, ^9^ Recognizing and managing these risk factors promptly is essential to improving patient outcomes and reducing hospital stay duration.^10^

The exact Fig.s vary depending on the study population, diagnostic methods, and definitions used, but the overall trend clearly indicates a substantial burden of this condition. Identifying the risk factors is crucial for developing effective prevention strategies. Predisposing factors, often inherent to the patient, may include advanced age, pre-existing cognitive impairment, frailty, sensory impairments (visual or auditory), and underlying comorbidities such as chronic kidney disease or heart failure.^3^^,^^4^^,^^11^ Precipitating factors, often related to the hospital environment or treatment, may include pain, dehydration, infection, medication side effects (especially from opioids or anticholinergics), metabolic disturbances, and sleep deprivation.^3^^,^^4^^,^^11^

Recent advances in machine learning (ML) have shown promise in addressing complex prognostic challenges in critical care.^11^ While ML models have been successfully applied to predict mortality in conditions like sepsis,^12^, ^13^, ^14^ their use in delirium, particularly for short-term mortality prediction, remains underexplored. This gap is significant given delirium's multifactorial nature and its distinct risk profile. Moreover, existing research often faces challenges such as limited model interpretability and reliance on imputed data, which may not fully reflect real-world clinical scenarios.

This study aims to develop and validate a machine learning-based predictive model to identify key risk factors for 28-day mortality among cancer patients with delirium admitted in Intensive Care Units. Five ML algorithms were evaluated for performance and generalizability to identify critical risk factors, with SHapley Additive exPlanations (SHAP) analysis enhancing model interpretability. By addressing the limitations of existing tools and prioritizing clinically actionable insights, this model seeks to enable early identification of high-risk patients, facilitating targeted interventions to reduce mortality and improve outcomes in this vulnerable population.

## Methods

### Study design

This retrospective cohort study examined risk factors associated with 28-day mortality in cancer patients diagnosed with delirium. Data were extracted from the Medical Information Mart for Intensive Care IV (MIMIC-IV) database, a publicly available, de-identified dataset. Structured query language (SQL) was used to retrieve relevant patient records and clinical variables.

### Participant selection

Patients were eligible for inclusion if they met the following criteria: (1) aged 18 years or older at the time of hospital admission; (2) confirmed diagnosis of cancer with delirium during hospitalization, identified by International Classification of Diseases, 10th Revision (ICD-10) code F05, OR screened positive for delirium using the ICU Assessment of Confusion of Consciousness (CAM-ICU); and (3) availability of complete 28-day mortality data following delirium diagnosis. Exclusion criteria were: (1) incomplete records for key variables, including mortality status or delirium diagnosis; (2) transfer to another facility before the 28-day endpoint, preventing mortality verification; and (3) pre-existing cognitive impairment or dementia prior to admission, to minimize confounding.

### Data source

Data were obtained from the MIMIC-IV database (version 3.0), which is a publicly available database developed by the MIT Computational Physiology Laboratory. It includes records of patients admitted to Beth Israel Deaconess Medical Center (https://mimic.physionet.org). MIMIC-IV (version 3.0) comprises comprehensive patient data, including demographics, vital signs, laboratory results, medications, procedures, and outcomes, covering the years 2008 through 2022. This study was conducted in accordance with the principles outlined in the Declaration of Helsinki. The MIMIC database adheres to an institutional review board-approved protocol in which all patient data are fully de-identified using randomly generated codes to protect individual privacy. The Ethics Committee of Sir Run Run Shaw Hospital granted an exemption from informed consent and ethical approval requirements for this secondary data analysis. The study received approval from the hospital's Institutional Review Board under Approval No. SYFYYLS2025Y#1035.

### Data extraction

Relevant variables were extracted from multiple tables within MIMIC-IV. The Admissions table provided hospital stay details, the Patients table provided demographic information, and the Diagnoses_icd table identified delirium cases. Laboratory results, such as creatinine and white blood cell counts, were retrieved from the Labevents table, while vital signs and clinical observations were sourced from the Chartevents table. Information on interventions, including mechanical ventilation and sedative use, was obtained from the Procedures_icd and Inputevents tables. SQL queries linked these tables using unique patient identifiers (subject_id, hadm_id), producing a unified dataset.

### Data preprocessing

Data preprocessing included handling missing data by excluding variables with > 20% missing values and imputing remaining missing values (≤ 20% missingness) using multiple imputation by chained equations (MICE) and k-nearest neighbors (KNN) methods. Continuous variables were then assessed for normality via histograms and Shapiro–Wilk tests, with non-normally distributed variables undergoing log-transformation or categorization. Derived variables were subsequently created, including the Sequential Organ Failure Assessment (SOFA) score to quantify organ dysfunction severity. Outliers, defined as values beyond three standard deviations from the mean, were removed to ensure data quality. Finally, all variables were standardized to enable feature comparability. To develop a predictive model for mortality in patients with delirium, the dataset was first split into a training set and a validation set using a 70:30 ratio. To address class imbalance, we applied the Synthetic Minority Over-sampling Technique (SMOTE). Unlike traditional oversampling, which simply duplicates minority class samples and may increase the risk of overfitting, SMOTE generates synthetic samples by interpolating between existing minority class instances in the feature space. This approach helps to balance the dataset while better preserving the original data distribution, thereby enhancing model generalizability and performance.

### Model construction

Five machine learning algorithms were developed to predict 28-day mortality, including category boosting (CatBoost), Decision Trees, Light Gradient Boosting Machine (LGBM), Logistic Regression, and Random Forest. Model development included feature optimization, hyperparameter tuning, and rigorous validation. We employed grid search for hyperparameter optimization, systematically exploring combinations of parameter values. By using cross-validation to identify the optimal configuration, this approach helps prevent the model from overfitting due to suboptimal parameter settings and enhances its generalizability. Receiver operating characteristic (ROC) curves validate machine learning models by assessing their classification accuracy and discrimination through the trade-off between true positive and false positive rates, summarized by area under the ROC curve (AUC), while calibration curves evaluate the reliability of predicted probabilities by comparing them to actual observed outcomes. DCA complements these by quantifying clinical utility through net benefit calculations across probability thresholds, guiding practical decision-making against strategies like treating all or none.

### Statistical analysis

Descriptive analysis was performed to present the basic demographic and clinical characteristics of cancer patients with delirium. Survival analysis methods included Kaplan–Meier curves with log-rank testing to compare cancer patients with delirium or without delirium, and multiple Cox proportional hazards regression models to evaluate the association between delirium and 28-day mortality in cancer patients. Model performance was assessed by the AUC to evaluate discrimination. Calibration was assessed using the Hosmer–Lemeshow goodness-of-fit test and calibration plots. Following a thorough assessment of model performance, we selected the final predictive model based on its superior stability across all evaluation metrics. To improve interpretability, this study employed SHAP, a game-theory-based method that quantifies feature contributions to individual predictions.^15^ This study applied SHAP analysis across multiple datasets to systematically evaluate, visualize, and interpret feature importance patterns. Model reliability was further validated through cross-validation, with both SHAP results and robustness checks providing granular insights into critical predictors and the model's generalizability. All statistical analyses were performed using DecisionLinnc software (v 1.0), R software (v 4.4.3) and Python software (v 3.8.5).

## Results

### The demographic and clinical characteristics of study sample

There were 1893 critical care patients with delirium who were included in the analysis, of whom 685 (36.2%) died within 28 days who were admitted to the intensive care unit at Beth Israel Deaconess Medical Center between 2008 and 2022. The basic characteristics and clinical information of cancer patients with delirium are presented in Table 1.

**Table 1:** Sociodemographic and clinical characteristics of cancer patients with delirium (*N* ​= ​1893).

Variables	Mean (SD)	*n* (%)
**Demographic**		
**Age, years**	69.92 (12.57)	
**Sex**		
Male		1136 (60.0)
Female		757 (40.0)
**Weight**	78.54 (21.48)	
**Height**	169.41 (10.58)	
**Medical insurance (*n* ​= ​1877)**		
Medicare		1179 (62.8)
Private		395 (21.0)
Medicaid		262 (14.0)
Other		41 (2.2)
**Marital status (*n* ​= ​1727)**		
Married		887 (51.4)
Single		478 (27.7)
Widowed		224 (13.0)
Divorced		138 (8.0)
**Laboratory and nursing indicators**		
WBC, ​× ​10^9^/L	17.14 (24.95)	
HB, g/L	9.40 (2.32)	
HCT, L/L	28.96 (6.91)	
PLT, ​× ​10^9^/L	178.64 (113.73)	
AST, U/L	379.37 (1457.16)	
ALT, U/L	179.85 (607.22)	
ALB, g/L	2.91 (0.72)	
BUN, mmol/L	33.43 (25.93)	
CA, mmol/L	8.40 (3.67)	
CR, μmol/L	1.63 (1.68)	
LAC, mmol/L	3.17 (2.61)	
Sodium, mmol/L	136.00 (5.99)	
Potassium, mmol/L	3.98 (0.64)	
Chloride, mmol/L	100.54 (6.73)	
Temperature, ^o^F	98.20 (95–100)	
SpO_2_, %	97.0 (95–100)	
**Clinical scoring**		
APACHE2	20.00 (4.12)	
SOFA	6.42 (3.90)	
SIRS	2.73 (0.87)	
APS III	54.60 (22.33)	
GCS	13.07 (8.92)	
**Ventilation**		
Yes		1631 (86.2)
No		262 (13.8)
Ventilation-hour	83.57 (115.95)	
ICU-day	6.44 (6.63)	
Hospital-day	17.20 (16.72)	
**Death within 28 days**		
Yes		685 (36.2)
No		1208 (63.8)
**Comorbidity, *n* (%)**	Yes	No
Myocardial infarct	241 (12.7)	1652 (87.3)
Congestive heart failure	411 (21.7)	1482 (78.3)
Peripheral vascular disease	167 (8.8)	1726 (91.2)
Cerebrovascular disease	319 (16.9)	1574 (83.1)
Chronic pulmonary disease	480 (25.4)	1413 (74.6)
Rheumatic disease	50 (2.6)	1843 (97.4)
Peptic ulcer disease	66 (3.5)	1827 (96.5)
Mild liver disease	292 (15.4)	1601 (84.6)
Diabetes without comorbidity	406 (21.4)	1487 (78.6)
Diabetes with comorbidity	187 (9.9)	1706 (90.1)
Paraplegia	167 (8.8)	1726 (91.2)
Renal disease	383 (20.2)	1510 (79.8)
Severe liver disease	175 (9.2)	1718 (90.8)

APACHE2, Acute Physiology and Chronic Health Evaluation II; APS3, Acute Physiology Score III; ALB, Albumin; ALT, Alanine transaminase; AST, Aspartate transaminase; BUN, Blood Urea Nitrogen; Ca, Calcium; CR, Creatinine; GCS, Glasgow Coma Scale; HB, Hemoglobin; HCT, Hematocrit; LAC, Lactate; PLT, Platelets; SIRS, Systemic inflammatory response syndrome; SOFA, Sequential Organ Failure Assessment; SpO_2_, Peripheral Oxygen Saturation; WBC, White blood cells.

### Relationship between delirium and 28-day mortality in cancer patients

**Fig. 1:**
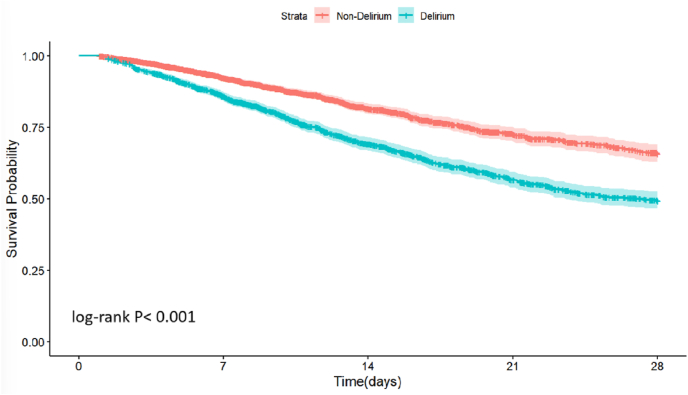
Kaplan–Meier survival curve analysis was used to compare 28-day mortality in cancer patients with or without delirium.

Based on whether cancer patients developed delirium, Kaplan–Meier survival curve analysis was drawn to compare the incidence of primary outcomes between the two groups with or without delirium (Fig. 1). The risk of 28-day mortality was significantly higher in cancer patients with delirium, with statistically significant differences (log-rank *P* ​< ​0.001).

In addition, the research team constructed five multifactor COX regression models for the relationship between delirium and the 28-day survival status of patients with sepsis (Table 2) to verify the stability of the above results. In model 1, without adjusting for any variables, results showed that cancer patients with delirium had a significantly increased risk of 28-day mortality (HR ​= ​1.80, 95% CI, 1.61–2.02, *P* ​< ​0.001) in the group without delirium. After adjusting for age and sex in Model 2, the analysis was very similar with the Model 1's results, but the degree of risk was slightly lower than

**Table 2:** Five multivariate COX regression models were constructed to study the 28-day mortality of cancer patients with delirium.

	Model 1 HR (95% CI)	Model 2 HR (95% CI)	Model 3 HR (95% CI)	Model 4 HR (95% CI)	Model 5 HR (95% CI)
**Delirium**					
**No**	Reference	Reference	Reference	Reference	Reference
**Yes**	1.80 (1.61–2.02)	1.75 (1.56–1.96)	1.71 (1.52–1.92)	1.35 (1.19–1.54)	1.69 (1.46–1.96)
***P*-value**	< 0.001	< 0.001	< 0.001	< 0.001	< 0.001

CI, confidence interval; HR, hazard ratio.

Model 1 was a non-adjusted model; Model 2 was adjusted for age (years), gender; Model 3 was adjusted based on the same parameters as Model 2 and made additional adjustments for comorbidities; Model 4 was adjusted using the same parameters as Model 3, with additional adjustments made to clinical scorings; Model 5 was adjusted using the same parameters as Model 4, with additional adjustments made for laboratory indicators.

In Model 1, and cancer patients in the delirium group still had a significantly increased risk of 28-day mortality (HR ​= ​1.75, 95% CI, 1.56–1.96, *P* ​< ​0.001). Model 3, Model 4, and Model 5 further adjusted for comorbidities, clinical scorings and laboratory indicators, the degree of risk of very consistent and the risk was HR ​= ​1.69 (95% CI, 1.46–1.96). Hence, by rigorously constructing and analyzing of several models, it is found that the trend is consistent with the results of Kaplan–Meier survival curve.

### Model performance comparisons

**Fig. 2:**
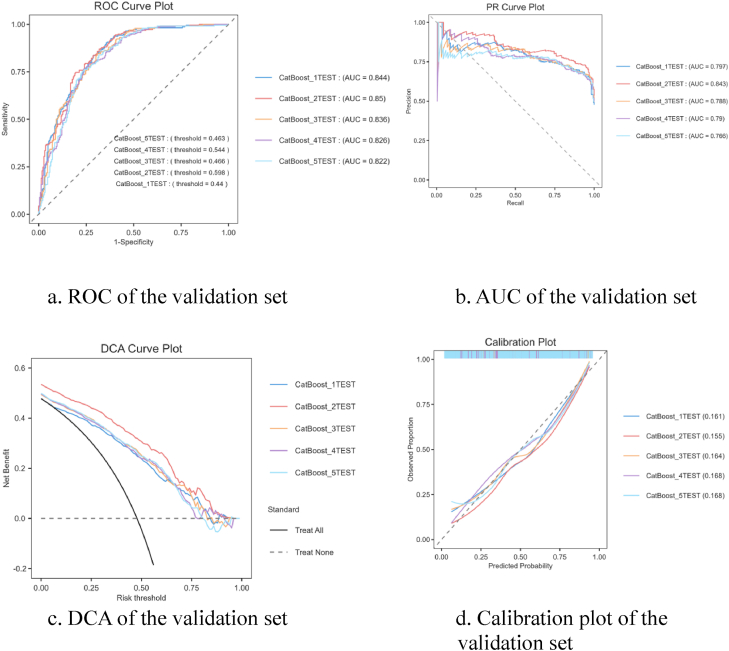
**The model performance of optimal method validation set by CatBoost model**. A: ROC of the validation set; B: AUC of the validation set; C: DCA of the validation set; D: Calibration plot of the validation set. AUC, area under the precision recall curve; CatBoost, categorical boosting; DCA, decision curve analysis; ROC, Receiver operating characteristic curve.

This study conducted a comprehensive analysis of the performance of five machine learning algorithms across various metrics on both the training and validation datasets. Findings were presented in Table 3. The CatBoost algorithm demonstrated relatively superior and stable performance on the training and validation sets, surpassing other algorithms across all metrics. Model performance was confirmed by the optimal validation set by CatBoost model. As shown in Fig. 2A–D, the CatBoost model achieves good ROC, AUC, DCA and calibration curve analysis. Fig. 2A and B indicate that the bias-corrected line of the CatBoost model closely aligned with the ideal line, reflecting strong agreement between predictions and observations. Additionally, DCA analysis revealed that CatBoost provided the greatest net benefit across threshold probabilities from 0 to 1 in both datasets (Fig. 2C and D). Consequently, CatBoost was selected as the optimal model for further interpretability analysis.

**Table 3:** Predictive performances of the five machine learning models.

Algorithm	Data set	AUC	Accuracy	Sensitivity	Specificity	Recall	F1 score
CatBoost	Train	0.979	0.916	0.934	0.899	0.934	0.917
	Validation	0.834	0.779	0.817	0.740	0.817	0.789
Decision tree	Train	0.832	0.765	0.839	0.688	0.839	0.784
	Validation	0.829	0.760	0.822	0.697	0.822	0.777
LGBM	Train	1	0.996	0.993	0.998	0.993	0.996
	Validation	0.824	0.763	0.795	0.731	0.795	0.773
Logistic regression	Train	0.800	0.709	0.669	0.748	0.669	0.695
	Validation	0.818	0.730	0.698	0.762	0.698	0.724
RF	Train	0.851	0.758	0.840	0.677	0.840	0.775
	Validation	0.831	0.765	0.839	0.688	0.839	0.784

CatBoost, categorical boosting; LGBM, Light Gradient Boosting Machine; RF, Random Forest; AUC, area under the curve.

### Feature importance by SHAP

**Fig. 3:**
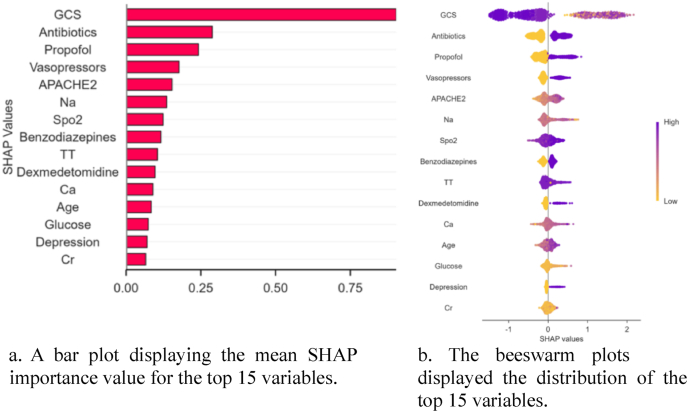
**The SHAP analysis of the CatBoost model**. A: A bar plot displaying the mean SHAP importance value for the top 15 variables; B: The beeswarm plots displayed the distribution of the top 15 variables. APACHE2, Acute Physiology and Chronic Health Evaluation II; Ca, Calcium; Cr, Creatinine; GCS, Glasgow Coma Scale; Na, Sodium; SpO_2_, Peripheral Oxygen Saturation; SHAP, SHapley Additive exPlanations; TT, temperature.

The CatBoost model was selected to predict 28-day all-cause mortality in cancer patients with delirium and to assess interpretability. SHAP analysis (Fig. 3A & B) revealed the most influential predictor that was Glasgow Coma Scale (GCS) scores (mean SHAP value ​> ​0.75), followed using antibiotics, propofol, vasopressors, and Acute Physiology and Chronic Health Evaluation II scores, all contributing significantly (SHAP value ​> ​0.2). Fig. 3A illustrates feature importance rankings based on mean absolute SHAP values, while Fig. 3B visualizes the directional relationship between feature values and their impact on predictions: the y-axis denotes importance ranking, and the x-axis reflects how increasing or decreasing feature values correlate with SHAP values (i.e., positive or negative contributions to mortality risk).

## Discussion

Due to very few studies conducted the prediction models among cancer patients with delirium, this study fills a notable gap in the literature by focusing specifically on mortality prediction in this vulnerable study population, a condition less explored compared to other critical illnesses such as sepsis.^13^ Among the five ML algorithms evaluated, the CatBoost model demonstrated superior performance, achieving good AUC on both training and validation datasets. The model's robustness was further evidenced by its excellent calibration, with the bias-corrected line closely aligning with the ideal line, and its provision of the greatest net benefit across all threshold probabilities in decision curve analysis.

Key predictors identified included GCS scores, the use of antibiotics, propofol, vasopressors, and Acute Physiology and Chronic Health Evaluation II scores, which emerged as the significant factors influencing mortality risk. These findings underscore the potential of the CatBoost model as a reliable tool for early risk stratification in this vulnerable population.^14^ To enhance interpretability, we incorporated clinical reasoning to contextualize key SHAP-identified predictors. Variables such as GCS, propofol and benzodiazepine use were categorized as delirium-specific, directly reflecting neurological status and pharmacological risks. In contrast, factors like antibiotic timing, laboratory blood test and respiratory parameters were considered non-specific contributors to overall disease severity. Findings from this study may shed light on identifying actionable targets for delirium-related interventions.

While previous research has leveraged ML to predict outcomes in sepsis patients, with models like XGBoost and LightGBM achieving AUCs of 0.90 or higher,^12^, ^13^, ^14^ the application to delirium has been limited. This study aligns with prior ML applications in critical care, such as sepsis mortality prediction, where CatBoost and SHAP have similarly excelled in performance and interpretability.^13^^,^^14^ Like these studies, our model emphasizes Acute Physiology and Chronic Health Evaluation II scores, reflecting shared pathways of organ dysfunction in critical illness. However, our focus on delirium introduces unique predictors, including propofol use - a modifiable risk factor specific to delirium management - highlighting the interplay between sedation practices and outcomes. While sepsis models prioritize coagulation and inflammatory markers (e.g., lactate, SOFA),^12^^,^^13^ this study underscores SpO_2_ and neurological assessments (GCS scores), bridging gaps in delirium-specific prognostication.

Although a high GCS score is typically associated with favorable outcomes, our findings reveal a contradictory pattern. A review of the existing literature revealed no prior studies documenting this specific clinical observation. We hypothesize that patients with higher baseline GCS scores at ICU admission may experience more significant GCS deterioration during delirium episodes, indicating the onset of acute and severe physiological insults during hospitalization. In contrast, patients with lower admission GCS scores may exhibit less fluctuation, possibly due to underlying chronic neurological conditions rather than new or worsening acute pathology. This unexpected trend highlights the need for further mechanistic studies to explore the relationship between baseline neurological status and delirium-related GCS changes.

Delirium often manifests as acute neuronal dysfunction and cerebral network disintegration, driven by systemic disturbances like inflammation (e.g., elevated cytokines including interleukin [IL]-1β, IL-6, and IL-8), neurotransmitter imbalances (e.g., reduced acetylcholine or altered serotonin and dopamine signaling), and oxidative stress, which collectively impair brain function and exacerbate underlying critical illnesses such as sepsis or trauma.^16^^,^^17^ A lower GCS score, indicative of diminished arousal and consciousness - a core feature of hypoactive delirium subtypes - reflects this neuronal dysregulation and is associated with heightened mortality risk due to secondary complications, including respiratory compromise, aspiration pneumonia, prolonged mechanical ventilation, and increased susceptibility to infections or thromboembolic events stemming from immobility and autonomic instability.^18^^,^^19^ These factors contribute to a dose–response relationship where the severity and duration of delirium, as captured by GCS, amplify systemic organ failure and long-term cognitive decline, thereby elevating overall mortality rates in critically ill populations.^20^

The strengths of this study include the use of the large, diverse MIMIC-IV database, which enhances the generalizability of our findings across varied cancer populations. This study utilized large dataset and found that the prevalence of delirium as high as 36.2% among cancer patients admitted to intensive care units, and found delirium is significantly related to high risk of mortality. In addition, good performance and interpretability of the CatBoost model, supported by SHAP analysis, provide a robust and actionable tool for clinical use.

However, limitations must be acknowledged. The retrospective design risks unmeasured confounding, and reliance on a single database may limit external validity. Exclusion of pre-ICU functional status and socioeconomic factors, which influence delirium outcomes, represents another constraint. ‌ The second limitation of this study is that the subtype classification method may have influenced the weights of predictive variables (e.g., strengthening the association between active delirium and sedative drug use), which could be addressed in future research by incorporating bedside assessment tools such as the DRS-R-98 to refine subtype data. Another limitation of this study is the lack of external validation. To enhance model reliability, we employed five-fold cross-validation. The validation results demonstrated that the model's AUC values remained consistently stable within the range of 0.822–0.850 (indicating a narrow fluctuation range of 0.028), confirming good predictive consistency and achieving a high level of overall accuracy.‌ Given the retrospective nature of our dataset and the limited availability of detailed clinical features required to reliably classify delirium subtypes, we were unable to conduct a valid stratified analysis. Future research should explore delirium subtypes as related to the mortality among cancer patients. Finally, this study acknowledged that it was not performed external validation, and suggesting future research should confirm the generalizability of our findings across diverse populations and settings.

Findings of this study underscore the potential of ML to enhance risk stratification in delirium, a condition historically challenging to prognosticate due to its multifactorial etiology. The clinical implications of this study are profound. By accurately predicting 28-day mortality in cancer patients with delirium, the CatBoost model enables early identification of high-risk individuals, facilitating timely and personalized interventions. For instance, the prominence of SpO_2_ as a predictor suggests that optimizing oxygenation could be a critical strategy to improve outcomes, prompting clinicians to closely monitor and manage respiratory status in these patients. The model's interpretability empowers healthcare providers with clear insights into the factors driving predictions, enhancing decision-making confidence. Potential integration into clinical decision support systems could offer real-time risk assessments, enabling proactive adjustments to treatment plans—such as intensifying monitoring, adjusting sedation, or initiating palliative care discussions for those at elevated risk. Ultimately, this tool has the potential to optimize resource allocation, reduce mortality, improve care quality for cancer patients with delirium across diverse critical care settings, and potentially reduce healthcare burdens in cancer delirium management.^21^

## Conclusions

This study developed an ML model using the MIMIC-IV database to predict 28-day mortality risk in cancer patients with delirium. The CatBoost model outperformed four other machine learning models. SHAP analysis effectively identified key risk factors influencing the mortality of cancer patients with delirium. These findings can aid clinicians in identifying high-risk cancer patients with delirium, optimizing medical resource allocation.

## CRediT authorship contribution statement

**Yang He, Ning Liu, Mimei Xu**: Conceptualization; **Yang He, Sicheng Hao**: Data sources & Data Analysis; **Mimei Xu, Yingchun Zeng**: Writing; All Authors: Review & Editing. All authors have read and approved the final manuscript.

## Ethics statement

This study received ethical approval from the Medical Ethics Committee of the Affiliated Hospital of Sir Run Run Shaw Hospital of Zhejiang University School of Medicine (Approval No. SYFYYLS2025Y#1035) and was conducted in accordance with the 1964 Helsinki Declaration and its later amendments or comparable ethical standards. All participants provided written informed consent.

## Data availability statement

The MIMIC-IV database is available from https://mimic.physionet.org.

## Declaration of generative AI and AI-assisted technologies in the writing process

No AI tools/services were used during the preparation of this work.

## Funding

This study was supported by the Medical Health Science and Technology Project of Zhejiang Provincial Health Commission (Grant No. 2025KY909, Yang He). The funders had no role in considering the study design or in the collection, analysis, interpretation of data, writing of the report, or decision to submit the article for publication.

## Declaration of competing interest

The authors declare no competing interests. The corresponding author, Dr. Yingchun Zeng, is an editorial board member of *Asia–Pacific Journal of Oncology Nursing*. The article was subject to the journal's standard procedures, with peer review handled independently of Dr. Zeng and their research groups.

## References

[1] Vonnes C., Tofthagen C.S. (2022). Impacting outcomes in the hospitalized oncology patient: evidence-informed quality and safety project to implement routine screening for delirium. Patient Safety.

[2] Ojiaku O.D.B., Duru P.C., Ikwuka A.O., Udeh F.C. (2024). Optimization of delirium care in adult patients with cancer: a comprehensive and integrative review of efficacy and patient outcomes. None.

[3] Seiler A., Blum D., Deuel J.W. (2021). Delirium is associated with an increased morbidity and in-hospital mortality in cancer patients: results from a prospective cohort study. Palliat Support Care.

[4] Arya Y., Syal A., Casipit C. (2024). Impact of delirium on outcomes among hospitalized patients with colorectal cancer: a United States population-based cohort study. J Clin Oncol.

[5] Nabi W., Javaid S., Ahmad S. (2024). Impact of delirium on in-patient outcomes in patients with pancreatic cancer: a nationwide study. J Clin Oncol.

[6] Hosie A., Phillips J., Lam L. (2020). A multicomponent nonpharmacological intervention to prevent delirium for hospitalized people with advanced cancer: a phase II cluster randomized waitlist controlled trial (The PRESERVE Pilot Study). J Palliat Med.

[7] Lei W., Ren Z., Su J. (2022). Immunological risk factors for sepsis-associated delirium and mortality in ICU patients. Front Immunol.

[8] Sadaf F., Saqib M., Iftikhar M., Ahmad A. (2023). Prevalence and risk factors of delirium in patients admitted to intensive care units: a multicentric cross-sectional study. Cureus.

[9] Varpaei H.A., Robbins L.B., Farhadi K., Bender C.M. (2024). Preoperative cognitive function as a risk factor of postoperative delirium in cancer surgeries: a systematic review and meta-analysis. J Surg Oncol.

[10] Quickfall D., Sklar M.C., Tomlinson G., Orchanian-Cheff A., Goligher E.C. (2024). The influence of drugs used for sedation during mechanical ventilation on respiratory pattern during unassisted breathing and assisted mechanical ventilation: a physiological systematic review and meta-analysis. eClinicalMedicine.

[11] Tao J., Seier K., Chawla S. (2024). Impact of delirium onset and duration on mortality in patients with cancer admitted to the ICU. J Intensive Care Med.

[12] Liu X., Niu H., Peng J. (2024). Improving predictions: enhancing in-hospital mortality forecast for ICU patients with sepsis-induced coagulopathy using a stacking ensemble model. Medicine (Baltim).

[13] Shen L., Wu J., Lan J., Chen C., Wang Y., Li Z. (2025). Interpretable machine learning-based prediction of 28-day mortality in ICU patients with sepsis: a multicenter retrospective study. Front Cell Infect Microbiol.

[14] Zhou S., Lu Z., Liu Y. (2024). Interpretable machine learning model for early prediction of 28-day mortality in ICU patients with sepsis-induced coagulopathy: development and validation. Eur J Med Res.

[15] Guo J., Cheng H., Wang Z., Qiao M., Li J., Lyu J. (2023). Factor analysis based on SHapley Additive exPlanations for sepsis-associated encephalopathy in ICU mortality prediction using XGBoost - a retrospective study based on two large database. Front Neurol.

[16] Maclullich A.M., Ferguson K.J., Miller T., de Rooij S.E., Cunningham C. (2008). Unravelling the pathophysiology of delirium: a focus on the role of aberrant stress responses. J Psychosom Res.

[17] Wilson J.E., Mart M.F., Cunningham C. (2020). Delirium. Nat Rev Dis Primers.

[18] Diwell R.A., Davis D.H., Vickerstaff V., Sampson E.L. (2018). Key components of the delirium syndrome and mortality: greater impact of acute change and disorganised thinking in a prospective cohort study. BMC Geriatr.

[19] Knox D.B., Lanspa M.J., Pratt C.M., Kuttler K.G., Jones J.P., Brown S.M. (2014). Glasgow Coma Scale score dominates the association between admission Sequential Organ Failure Assessment score and 30-day mortality in a mixed intensive care unit population. J Crit Care.

[20] Ormseth C.H., LaHue S.C., Oldham M.A., Josephson S.A., Whitaker E., Douglas V.C. (2023). Predisposing and precipitating factors associated with delirium: a systematic review. JAMA Netw Open.

[21] Coolens O., Kaltwasser A., Melms T. (2025). Delirium management in 2024: a status check and evolution in clinical practice since 2016. Intensive Crit Care Nurs.

